# A dataset of topographic correction coefficients for shortwave downward radiation over the Pan-Third Pole

**DOI:** 10.1038/s41597-024-03616-2

**Published:** 2024-07-16

**Authors:** Yuyang Xian, Tianxing Wang, Yihan Du, Husi Letu, Jianbo Qi, Dahui Li

**Affiliations:** 1 School of Geospatial Engineering and Science, Sun Yat-sen University & Southern Marine Science and Engineering Guangdong Laboratory (Zhuhai), Zhuhai, 519000 China; 2 Key Laboratory of Comprehensive Observation of Polar Environment (Sun Yat-sen University), Ministry of Education, Zhuhai, 519000 China; 3 Key Laboratory of Natural Resources Monitoring in Tropical and Subtropical area of South China, Ministry of natural resources, Zhuhai, 519000 China; 4 Guangdong Provincial Key Laboratory of Geodynamics and Geohazards, School of Earth Sciences and Engineering, Sun Yat-Sen University, Zhuhai, 519000 China; 5 State Key Laboratory of Remote Sensing Science, Aerospace Information Research Institute, Chinese Academy of Sciences, Beijing, 100094 China; 6 State Key Laboratory of Remote Sensing Science, Faculty of Geographical Science, Beijing Normal University, Beijing, 100875 China

**Keywords:** Geography, Atmospheric science

## Abstract

Prevalent Shortwave downward radiation (SWDR) estimates assume a flat surface, neglecting topographic effects and leading to significant errors in mountainous regions. We introduce SWDR topography correction coefficients (TCCs), based on the mountain radiative transfer model tailored for the Pan-Third Pole region. This dataset effectively bridges the disparities between flat-surface SWDR and rugged-surface SWDR, forming part of the Long-term Earth System spatiotemporally Seamless Radiation budget dataset (LessRad). Validation results using a three-dimensional radiative transfer model demonstrate the efficacy of this method in correcting solar direct radiation, sky diffuse radiation, and SWDR under diverse conditions. At a spatial resolution of 2.5 arc-minutes, the correction accuracy for solar direct radiation is characterized by a coefficient of determination (R²) of 0.998, a relative root mean square error (rRMSE) of 2.4%, and a relative bias (rbias) of 0.8%. For sky diffused radiation, an R² of 0.965, a rRMSE of 1.2%, and a rbias of −0.8%. SWDR corrections under clear and cloudy skies also show high accuracy, demonstrating the robustness of the TCCs approach.

## Background & Summary

Shortwave downward radiation (SWDR) constitutes a fundamental component in the Earth’s material and energy balance, carrying significant implications for global climate dynamics and biological processes^1–4^. SWDR variations exert direct and far-reaching effects, influencing surface temperature, humidity, and energy fluxes^2,5^. Various methods are currently used for SWDR estimation, categorically divided into ground-based observations, numerical simulations, data reanalysis, and satellite remote sensing inversions^6,7^. In recent decades, significant progress has been made in SWDR inversion methods and their applications, driven by the continuous efforts of scholars. And they provide various global-scale, high spatial and temporal resolution SWDR data products, including notable sources like Clouds and the Earth’s Radiant Energy System (CERES)^8,9^, the European Centre for Medium-Range Weather Forecasts (ECMWF) next-generation reanalysis (ERA5)^10^, MCD18^11^, Breathing Earth System Simulator (BESS)^12,13^, and the Global Land Surface Satellite (GLASS)^5,14^.

Currently, only a few of SWDR products account for terrain influence, exemplified by the SWDR derived by Letu *et al*.^6^ using Himawari-8/AHI inversion. However, the remote sensing data offered by Letu *et al*.^6^ is constrained to a limited temporal span, and its overall accuracy may not always surpass that of existing datasets. As exemplified by the SWDR products discussed in the previous paragraph, most SWDR datasets were generated under the premise of a flat surface, neglecting the influence of terrain (such as slope, aspect, and shadow). Complex terrain areas suffer from the limitations of this flat surface assumption, resulting in substantial errors in SWDR estimations within mountainous regions, and inappropriately smoothing the spatial distribution of SWDR^15,16^. Mountainous regions exhibit significant spatiotemporal heterogeneity in SWDR, influenced by factors such as slope, aspect, and shading effects^17,18^. Existing topographic correction methods of SWDR in mountainous terrains primarily fall into two categories: mountain radiative transfer models (MRTM)^15,16,19–22^ and machine learning approaches^23–26^. Among these, MRTM describes the complex interaction between SWDR and the surface, facilitating the derivation of SWDR values actually received by mountainous terrain. In contrast to machine learning, MRTM offers an advantageous combination of specific physical principles and high terrain correction accuracy, albeit at the cost of computational intensity and operational inefficiency^27^. The utilization of MRTM for terrain correction of planar-assumed SWDR necessitates a sequence of scale conversion procedures. Specifically, MRTM is devised at the Digital Elevation Model (DEM) scale, typically with a spatial resolution of meter-level, while common SWDR datasets boast spatial resolutions of kilometer-level. Consequently, it becomes imperative to initially downscale the SWDR outcomes to match the spatial resolution of the DEM, employing techniques such as resampling, to drive the MRTM for terrain correction. Subsequently, these downscaled results need to be re-aggregated to their original kilometer-level spatial resolution. The scale conversion process demands substantial computing resources and is notably time-intensive. Moreover, we have observed ongoing optimization efforts in methods for estimating SWDR, particularly enhancing accuracy on flat surfaces. Simultaneously, the correlation between SWDR under assumption of a flat surface and it received on actual mountainous surfaces is primarily contingent on terrain characteristics and remains unaffected by alterations in data sources and inversion methodologies. Consequently, we propose the Topography Correction Coefficients (TCCs) dataset as a pivotal intermediary to facilitate the application of various SWDR results inverted under the assumption of a flat surface in mountainous regions. And it serves as a topography correction coefficients product within the Long-term Earth System spatiotemporally Seamless Radiation budget dataset (LessRad).

In the Pan-Third Pole region, with the Tibetan Plateau as its center, stands as one of the world’s most complex terrestrial environments, significant terrain variation has profound effects on SWDR^28^. To facilitate effective SWDR terrain correction within this intricate region using MRTM, this study has introduced a multi-spatial scale terrain correction parameterization product based on Shuttle Radar Topography Mission (SRTM) data. The SRTM, conducted by NASA, aimed to obtain high-precision three-dimensional terrain data of the Earth’s surface using radar imaging technology deployed on the space shuttle. This parameterization dataset covers spatial resolutions of 3 arc-seconds, 15 arc-seconds, 30 arc-seconds, 1 arc-minute, and 2.5 arc-minutes. It comprehensively addresses the impact of varying solar positions and terrain conditions on SWDR, encompassing direct radiation and sky diffuse radiation. The result is a transformative process that converts original SWDR obtained under the flat surface assumption into the terrain-corrected SWDR considered the effect of mountainous surfaces.

The accuracy of terrain-corrected SWDR depends on two critical factors: the chosen terrain correction method and the accuracy of the original SWDR data assumed under the flat surface. Several considerations prompted our decision to construct a comprehensive SWDR terrain correction coefficient dataset, rather than directly providing SWDR results after terrain correction. (1) With the continual advancement of remote sensing technology and inversion algorithms, there are improvements in spatial and temporal resolutions of SWDR products, as well as their accuracy. (2) MRTM have showed exceptional accuracy in SWDR correction. Simultaneously, the accuracy of Digital Elevation Model (DEM) data remains reliably stable, with minor improvements in DEM data accuracy having minimal influence on the outcomes of terrain correction. (3) In contrast to the variety of available SWDR products, terrain-corrected SWDR products remain notably scarce. The SWDR terrain correction parameterization defined in this study is established as the ratio between SWDR received by the rugged surface and SWDR received under the assumption of a flat plane. This ratio is inherently linked to the terrain characteristics and remains unchanged regardless of variations in incident radiation magnitude. Consequently, for SWDR products obtained under the flat surface assumption, at any given moment and through any method, such SWDR terrain correction coefficient products serve as a versatile tool for efficient terrain correction implementation. By constructing multi-spatial scale TCCs dataset, we streamline the terrain correction process for data users, enabling them to select TCCs results with the same spatial resolution as the SWDR data. Through simple processing, users can obtain terrain-corrected SWDR results in mountainous areas. This approach not only ensures reliable results but also significantly reduces the computational burden on data users. Our overarching goal is to offer support to current and future SWDR researchers in mitigating the uncertainties arising from terrain effects when applying their algorithms to mountainous regions. This TCCs datasets are not only applicable to various existing SWDR datasets without terrain correction but also to potentially higher-precision SWDR results under future flat surface assumptions.

## Methods

### Study area

**Fig. 1:**
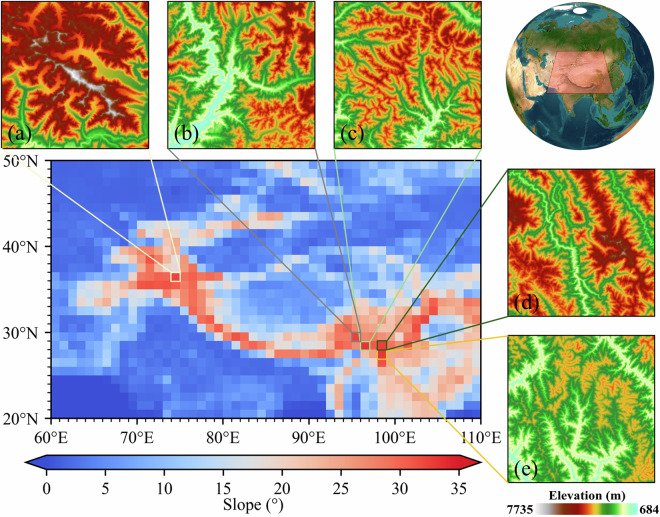
The average slope distribution of the Pan-Third Pole region and five 1°x1° sample areas.

This study primarily focuses on the Tibetan Plateau and its neighbouring regions, located within the geographic coordinates of 20°-50°N and 60°-110°E, encompassing Central Asia—an area colloquially referred to as the Pan-Third Pole (Fig. 1). This extensive region holds the distinction of being the largest expanse of the glacier and snow-covered terrain outside the polar regions. It serves as a critical reservoir of freshwater resources and is renowned as the ‘Asia Water Tower’^29,30^. This geographical feature plays a pivotal role in the context of global climate dynamics, carbon equilibrium, biodiversity preservation, and various other ecological factors^31–34^. Simultaneously, its intricate and multifaceted surface morphology exerts a profound influence on the spatial distribution of SWDR at the Earth surface^15,16^. To better evaluate our research, we analyzed the slope characteristics in the Pan-Third Pole region and identified the five 1°x1° areas with the highest average slope as sample demonstration areas, as outlined in Fig. 1 and Table 1.

**Table 1:** Location and topographic information of the five sample areas.

	Label	Latitude	Longitude	DEM range	Average slope	Maximum slope
a	N36E074	36–37°N	74–75°E	3234–7735 m	32.342°	80.9404°
b	N29E095	29–30°N	95–96°E	684–5406 m	32.3361°	86.1018°
c	N28E096	28–29°N	96–97°E	1344–5186 m	33.3478°	85.4043°
d	N28E098	28–29°N	98–99°E	1698–6471 m	32.3898°	83.1256°
e	N27E098	27–28°N	98–99°E	1388–4166 m	33.0917°	81.5754°

### Source data

The Shuttle Radar Topography Mission (SRTM) is a collaborative undertaking involving the National Aeronautics and Space Administration (NASA), the National Geospatial-Intelligence Agency (NGA), and the German and Italian Space Agencies. Its primary objective was to acquire high-resolution terrain data concerning the Earth surface. SRTM successfully generated a digital elevation model (DEM) covering a substantial portion of the Earth’s surface, ranging from 60°N to 56°S. The SRTM DEM dataset is distinguished by its data accuracy and offers two key resolutions: SRTM1, featuring a horizontal resolution of 1 arc-second, and SRTM3, with a horizontal resolution of 3 arc-seconds^35–37^. For the Pan-Third Pole region, SRTM1 DEM data was obtained through the Google Earth Engine (GEE) platform^35,38,39^. Furthermore, calculations of slope and aspect were conducted and corresponding datasets were acquired through the GEE platform.

#### Method flow

**Fig. 2:**
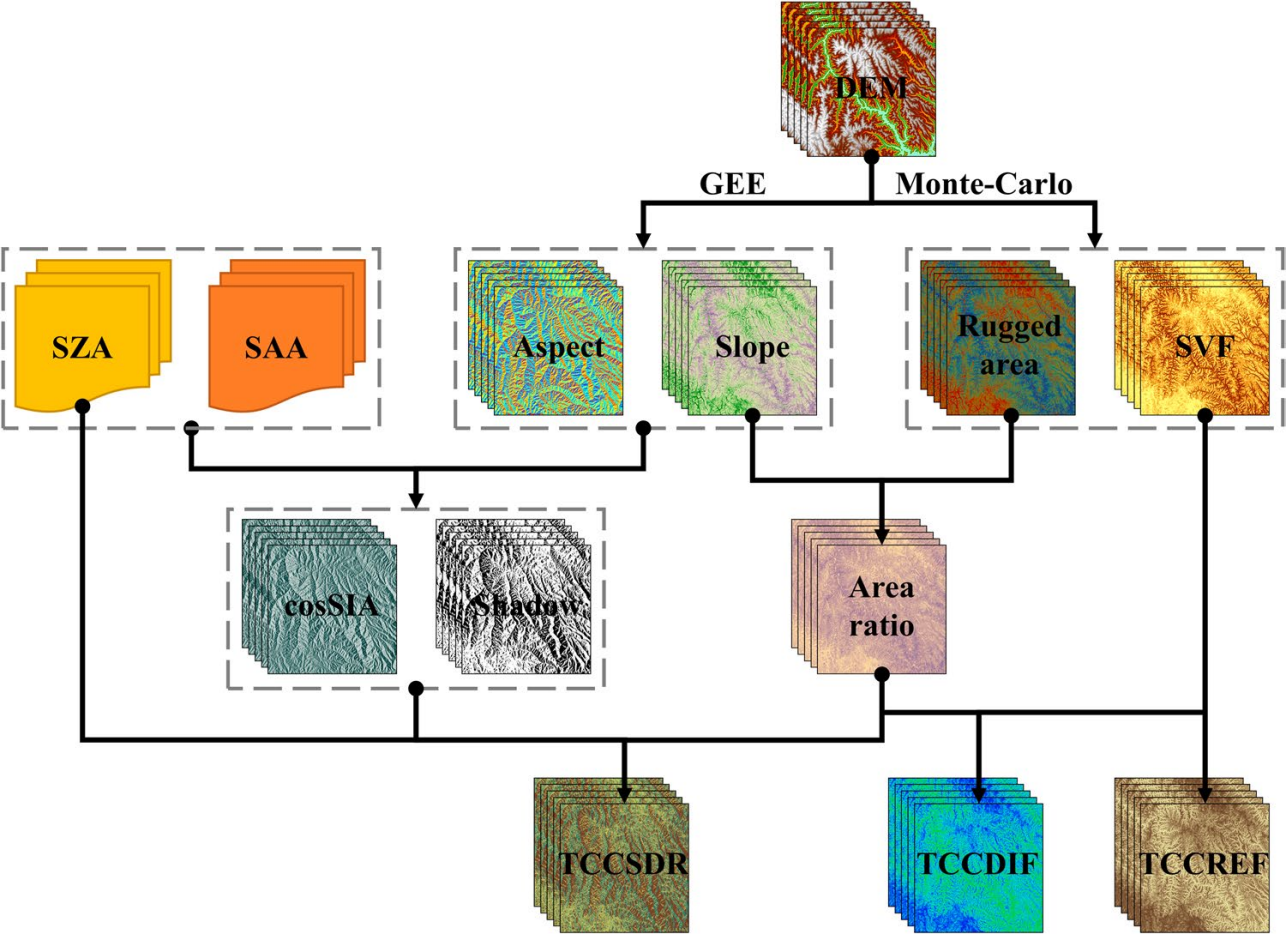
Flow chart of SWDR topography correction coefficient dataset. Where, the SZA is solar zenith angle; the SAA is solar azimuth angle; the SVF is the sky visibility factor; the cosSIA is the cosine of the solar incident angle; the TCCSDR is terrain correction coefficient for solar direct radiation; the TCCDIF is terrain correction coefficient for diffuse radiation; the TCCREF is terrain correction coefficient for adjacent surface reflected radiation.

This study builds upon the Uniform ShortWave Topographic Radiation Model (USWTRM) method, established by Xian^16^, and undertakes a comprehensive and detailed investigation of the terrain coefficient calculation process. The flow chart for obtaining terrain correction coefficient data for surface SWDR in the Pan-Third Pole region is depicted in Fig. 2.

### The calculation of sky visibility factor

The Sky Visibility Factor (SVF) serves as a valuable metric for quantifying the proportion of the visible sky within the hemispheric space^40^, and it can be expressed as Eq. (1).

1 $${SVF}\approx \frac{1}{2\pi }{\int }_{0}^{2\pi }[{\cos }({Slope}){{\sin }}^{2}{H}_{\phi }+{\sin }({Slope}){\cos }(\phi -{Aspect})({H}_{\phi }-{\sin }{H}_{\phi }{\cos }{H}_{\phi })]{d}_{\phi }$$

where, ${H}_{\phi }$ denotes the horizon angle for each direction $\phi$. If there is no horizontal occlusion in direction $\phi$, then ${H}_{\phi }=\pi /2$.

This coefficient holds significant importance in assessing the degree of openness or exposure of a given area to the sky and is particularly critical for the accurate quantification of diffuse radiation received by mountainous terrains. To achieve this, we employ DEM data to accurately determine the SVF for each pixel on the mountainous surface through a Monte Carlo simulation methodology. Specifically, our approach uses the target pixel as the central reference point, and systematically evaluating the occlusion of the target pixel within the visible sky range by neighbouring pixels at 16 azimuth angles. This process allows for the accurate computation of the SVF. It is worth noting that in the estimation of SVF using the Monte Carlo method, the selection of the search radii is a pivotal parameter, closely linked to the overall accuracy of the results^41^. To identify the search radii, we tested the SVF results under different search radii in five sample areas with the largest average slopes in Fig. 1.

**Fig. 3:**
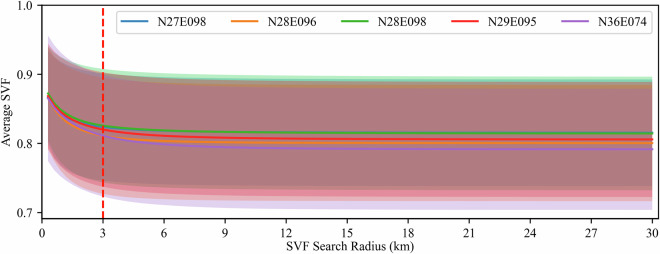
The average SVF calculated by using different search radius in Monte Carlo simulation.

Following tests in five representative sample areas (Fig. 3), we ultimately determined the search radii to be 3 km. It is worth noting that the 3 km distance is derived by converting the DEM data from the geographical coordinate system to the projected coordinate system. In the geographical coordinate system, this distance is expressed as 100 arc-seconds. This selection offers a favourable balance between computational efficiency and result accuracy. Moreover, while longer search radii allow for the consideration of potential occlusions by more distant mountains, they also entail stronger path absorption and scattering of radiation by the atmosphere during transmission. Consequently, radiation changes induced by distant mountain occlusions become more susceptible to atmospheric influence, thereby introducing additional uncertainty. Furthermore, these search radii align with the prevailing spatial resolution of commonly used SWDR algorithms^6,12,42,43^, with a spatial resolution of around 5 km. As such, the selected search radii comprehensively capture the influence of local topography on the sky’s visible range within each coarse pixel.

### The calculation of shadow

Direct radiation uniformly illuminates all points on a flat surface, but in mountainous areas, it is highly susceptible to shadows caused by terrain obstacles. These shading phenomena encompass two primary categories: self-shading and shading induced by neighbouring mountains. To address this, we employ the Monte Carlo simulation method to calculate mountain shadows under varying solar zenith angles (SZA) and solar azimuth angles (SAA). Similar to the SVF, the calculation outcomes for shadow are influenced by the chosen search radii^44^. Therefore, we employ the same search radii as used for SVF. In the simulation, SZAs and SAAs are systematically sampled at 5° intervals. This approach yields shadow results for a range of SAZ and SAA combinations, comprehensively capturing the diverse scenarios of shading across mountainous landscapes.

### The calculation of solar incidence angle

The solar direct radiation reaching the surface of mountainous terrain is governed by the solar incident angle. This angle is conventionally defined as the angle between the incoming solar rays and the perpendicular line to the slope. It is frequently expressed in the form of its cosine (denoted as ${\cos }{SIA}$). Smaller solar incident angles, corresponding to larger values of ${\cos }{SIA}$, result in a higher intensity of solar direct radiation per unit area received by the Earth’s surface. The ${\cos }{SIA}$ is intricately linked to factors such as slope, aspect, SZA, and SAA. Its specific calculation formula is as follows:

2 $${\cos }{SIA}={\cos }\left({SZA}\right){\cos }\left({slope}\right)+{\sin }\left({SZA}\right){\sin }\left({slope}\right){\cos }\left({SAA}-{aspect}\right)$$

3 $${\cos }{SIA}=\left\{\begin{array}{l}{\cos }{SIA}({\cos }{SIA} > 0)\\ 0\,({\cos }{SIA}\le 0)\end{array}\right.$$

when ${\cos }{SIA}$ is greater than 0, it indicates that the Earth’s surface is receiving solar direct radiation. Conversely, when ${\cos }{SIA}$ equals 0, it signifies that the ground surface is not receiving solar direct radiation due to self-shading or because the sun is positioned below the horizon.

### The calculation of rugged surface area

**Fig. 4:**
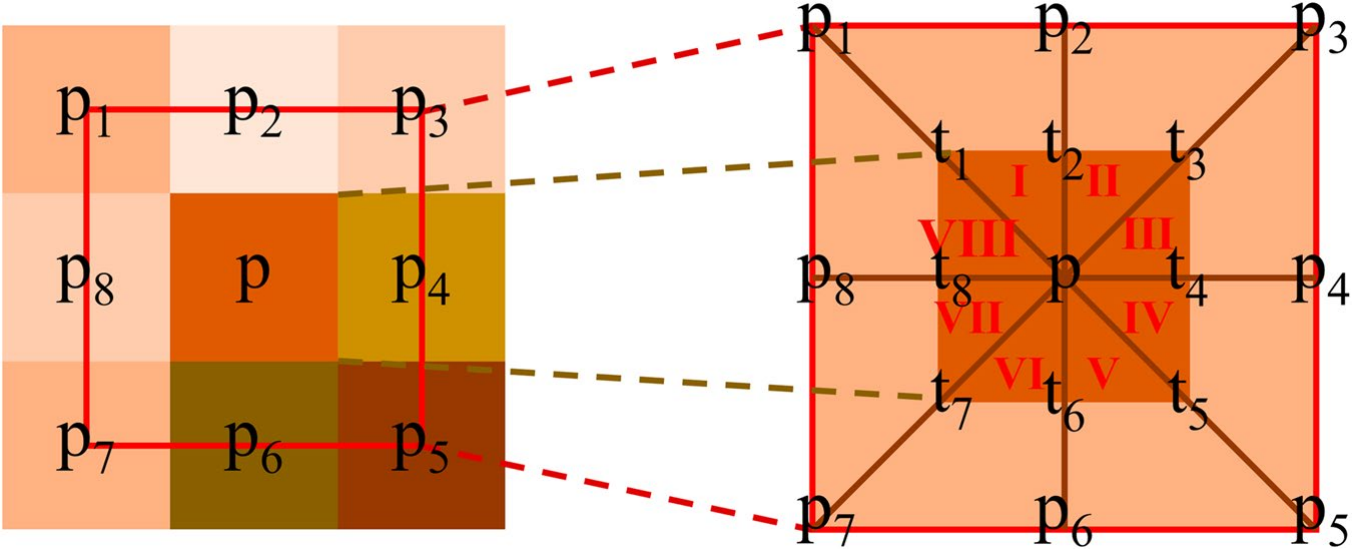
Surface area calculation based on irregular triangles.

Undulating terrains within mountainous regions often result in a surface area that is substantially larger than its projected area. This discrepancy in surface area has a direct impact on the irradiance (measured in W/m²) received by the mountainous surface. To calculate the rugged surface area for an DEM pixel, we utilize data from eight neighbouring DEM pixels. Specifically, this process involves the creation of eight irregular triangles (labelled I to VIII) connecting the central point of the target DEM pixel with the central points of the surrounding eight DEM pixels. Subsequently, the area of each of these triangles is calculated, and the sum of the areas falling within the domain of the target DEM pixel is determined^45^ (Fig. 4). Its calculation formulas are as follows:

4 $${S}_{p}={S}_{I}+{S}_{{II}}+{S}_{{III}}+\ldots +{S}_{{VIII}}$$

${S}_{p}$ represents the surface area of the target DEM pixel $p$, which is comprised of the area of eight triangles formed by the connection of the target pixel with eight neighbouring DEM pixels.

As an example, the area of the three-dimensional ${S}_{I}$, formed by the $p$, ${t}_{1}$, and ${t}_{2}$, is determined through the utilization of Heron’s Formula:

5 $${S}_{{\rm{I}}}=\sqrt{s\left(s-a\right)\left(s-b\right)\left(s-c\right)}$$

6 $$s=\frac{a+b+c}{2}$$

7 $$a={L}_{t1-t2}=\frac{{L}_{p1-p2}}{2}=\frac{\sqrt{{\left({H}_{p1}-{H}_{p2}\right)}^{2}+{S}_{h}}}{2}$$

8 $$b={L}_{t1-p}=\frac{{L}_{p1-p}}{2}=\frac{\sqrt{{\left({H}_{p1}-{H}_{p}\right)}^{2}+2\times {S}_{h}}}{2}$$

9 $$c={L}_{t2-p}=\frac{{L}_{p2-p}}{2}=\frac{\sqrt{{\left({H}_{p2}-{H}_{p}\right)}^{2}+{S}_{h}}}{2}$$

Where, ${H}_{p}$, ${H}_{p1}$, and ${H}_{p2}$ are the elevation of DEM pixel p, p1 and p2, respectively. And ${S}_{h}$ is the projected area of the DEM pixel.

### The calculation of area ratio

To quantify the distinction between the actual surface area of mountainous regions and their projected planar area, we employ the area ratio factor ($t$) for measurement.

10 $$t=\frac{{S}_{h}}{{\cos }\left({slope}\right)\times {S}_{p}}$$

In theory, $t$ should not exceed 1. However, in regions characterized by extremely steep terrains, significant slopes result in $\cos \left({slope}\right)$ approaching 0. Under such circumstances, even minor inaccuracies in slope calculations can disproportionately impact $t$ potentially leading to anomalous situations where $t > 1$. To mitigate the influence of outliers and reduce the error, we constrain $t\in (\mathrm{0,1}]$ (Eq. 11).

11 $$t=\left\{\begin{array}{c}t\left(t\le 1\right)\\ 1\left(t > 1\right)\end{array}\right.$$

### The calculation of terrain correction coefficient

After building upon the terrain calculations described above, we are equipped to compute the terrain correction coefficients (TCCs) at a 1 arc-second resolution for the three components of SWDR, namely: TCCSDR (Terrain Correction Coefficient for Solar Direct Radiation), TCCDIF (Terrain Correction Coefficient for Sky Diffuse Radiation), and TCCREF (Terrain Correction Coefficient for Adjacent Surface Reflected Radiation)^15,16,19–22^.

In mountainous areas, solar direct radiation is influenced by factors such as the solar zenith angle, solar incident angle, shadow, and area ratio^16,21^. Consequently, the TCCSDR can be calculated using Formula 12. And the SVF represents the proportion of the sky hemisphere observed by surface pixels in mountainous regions. Hence, under the single slope assumption, SVF serves as a conversion coefficient for the sky diffuse radiation received by mountainous surfaces compared to flat surfaces^16,40^. When considering the shape of the mountainous surface, the TCCDIF can be described by Formula 13. In parallel to SVF, the adjacent terrain reflections received by mountainous surfaces originate from the portion of the hemisphere obstructed by terrain. Therefore, in estimating reflected radiation from mountainous terrain, the complement of SVF is often utilized as a parameter to quantify the radiation characteristics of this segment^20,46^. This definition is also applied to TCCREF, which is described by Formula 14.

12 $${\rm{T}}{\rm{CCSDR}}=\frac{{\cos }{SIA}\times \varTheta }{{\cos }\left({SZA}\right)}\times t$$

13 $${\rm{TCCDIF}}={SVF}\times t$$

14 $${\rm{TCCREF}}=1-{SVF}$$

Where, $\varTheta$ represents the mountain shading factor, which is 0 when the pixel is in the shadow and 1 when it is not shaded.

Following the methodology outlined above, we initially obtained SWDR TCCs at a fine 1 arc-second spatial resolution, which corresponds to the resolution of the DEM. Subsequently, we conducted spatial upscaling on these high-resolution results for two major reasons. Firstly, when compared to coarser spatial scales, TCCs at the 1 arc-second spatial resolution exhibit a heightened sensitivity to potential data production errors^47^. Enhancing the spatial scale of these TCCs serves to mitigate the impact of errors on the terrain correction results. Secondly, many widely used SWDR products, such as MODIS, Himawari-8, GLASS, and others, feature relatively coarse spatial resolutions^5,6,10,14,43,48,49^. Adapting the TCCs products to match these coarser spatial scales offers the advantage of facilitating the direct applicability of the data.

As a result, we aggregated the initially obtained 1 arc-second TCCs. This process yielded datasets at various resolutions, including 3 arc-seconds (approximately 100 m), 15 arc-seconds (about 500 m), 30 arc-seconds (approximately 1 km), 1 arc-minute (approximately 2 km), and 2.5 arc-minutes (approximately 5 km). These datasets are georeferenced in the WGS1984 coordinate system.

### Data accuracy validation based on the three-dimensional radiative transfer model

The evaluation of SWDR accuracy faces notable challenges in mountainous regions. Firstly, there is a lack of observations from stations located on mountainous slopes. Secondly, the spatial distribution of SWDR in mountainous areas is heavily influenced by terrain variations, resulting in a high degree of spatial heterogeneity. Consequently, accurately assessing the accuracy of surface-scale data using point-scale observations is a formidable task^50^. Thirdly, terrain-corrected SWDR outcomes are not only influenced by TCCs but are also significantly reliant on the accuracy of the input SWDR data under the flat surface assumption.

In this context, the challenge lies in establishing a robust methodology for assessing the accuracy of TCCs. Fortunately, the three-dimensional radiative transfer model provides a valuable bridge to furnish a theoretical reference, enabling the evaluation of the accuracy of TCCs. LESS (large-scale remote sensing data and image simulation framework) is a ray-tracing based three-dimensional radiative transfer model capable of achieving rapid, efficient, and accurate simulations of radiative transfer over large regions^51,52^. Within LESS, we employ various scenarios representing different solar incidence angles and surface states to simulate multiple sets of SWDR received by the surface (Table 2). These simulated results are then compared to the correction outcomes achieved through TCCs. It is worth noting that both this study and LESS ignored the anisotropy of diffuse radiation.

**Table 2:** Input conditions for LESS.

Parameter	Clear-sky	Cloudy-sky
Incident solar direct radiation	1367 W/m^2^	0
Incident diffuse radiation	0	1000 W/m^2^
SZA	20, 45, 70	—
SAA	90	—
Albedo (Lambertian)	0, 0.2 (Grass), 0.8 (Snow)	
Photon density	25/m^2^	

By combining the SWDR acquired through the flat surface setting in LESS with TCCs dataset, the terrain-corrected SWDR for the five test areas can be readily computed, as follows:

15 $${E}_{{SDR}}={\rm{T}}{\rm{CCSDR}}\times {E}_{{SDR}}^{H}$$

16 $${E}_{{DIF}}={\rm{T}}{\rm{CCDIF}}\times {E}_{{DIF}}^{H}$$

17 $${E}_{{REF}}=\frac{{\rm{T}}{\rm{CCREF}}\times \left({E}_{{SDR}}+{E}_{{DIF}}\right)\times \bar{\rho }}{1-{\rm{T}}{\rm{CCREF}}\times \bar{\rho }}$$

18 $${E}_{{SWDR}}={E}_{{SDR}}+{E}_{{DIF}}+{E}_{{REF}}$$

Where, ${E}_{{SWDR}}$, ${E}_{{SDR}}$, ${E}_{{DIF}}$, and ${E}_{{REF}}$ represent the SWDR, solar direct radiation, diffuse radiation, and reflected radiation from adjacent pixels, which are actually radiation flux density received by the mountainous surface. ${E}_{{SDR}}^{H}$ denotes the direct radiation received by a horizontal surface and is equivalent to the Incident solar direct radiation multiplied by the cosine of the SZA. ${E}_{{DIF}}^{H}$ represents the diffuse radiation received by the horizontal surface and is equivalent to Incident diffuse radiation. $\bar{\rho }$ stands for the average surface albedo of the target pixel and its adjacent pixels. In the LESS simulation, the ground surface is modelled as Lambertian with a consistent reflectivity, and thus, $\bar{\rho }$ aligns with the surface albedo.

### Statistical metrics

We have chosen three statistical metrics to assess the effectiveness of the dataset for SWDR terrain correction: coefficient of determination ${R}^{2}$, relative root mean square error (rRMSE), and relative bias (rbias).

Coefficient of determination (${R}^{2}$)

19 $${R}^{2}=\frac{\mathop{\sum }\limits_{i=1}^{M}({P}_{i}-\bar{P})({W}_{i}-\bar{W})}{\left[\mathop{\sum }\limits_{i=1}^{M}{({P}_{i}-\bar{P})}^{2}\,\mathop{\sum }\limits_{i=1}^{M}{({W}_{i}-\bar{W})}^{2}\right]}$$

Relative root-mean-square error (rRMSE)

20 $${rRMSE}=|\frac{\sqrt{\mathop{\sum }\limits_{i=1}^{M}\frac{{({{P}_{i}-W}_{i})}^{2}}{N}}}{\bar{W}}|\times 100 \% $$

Relative bias (rbias)

21 $${rbias}=\left|\frac{\frac{\mathop{\sum }\limits_{i=1}^{M}\left({P}_{i}-{W}_{i}\right)}{N}}{\bar{W}}\right|\times 100 \% $$

Where, ${W}_{i}$ is the SWDR simulated using the LESS model under different mountain surface conditions; ${P}_{i}$ denote the result of Eqs. 15–18; $i=1,2,3\ldots ,N$ (where *N* is the total number of sample points selected in the study region); and $\bar{W}$ and $\bar{P}$ are the average of ${W}_{i}$ and ${P}_{i}$, respectively. ${R}^{2}$ is used to evaluate the closeness of the corresponding sample points. The ${rRMSE}$ is often used to measure the extent of the average error. The rbias indicates the systematic error between two models, which is usually described by overestimation and underestimation.

## Data Records

Leveraging SRTM data, we have devised a multi-spatial scale terrain correction parameterization product specifically tailored for the Pan-Third Pole region, encompassing spatial resolutions of 3 arc-seconds, 15 arc-seconds, 30 arc-seconds, 1 arc-minute, and 2.5 arc-minutes. The Pan-Third Pole region shortwave downward radiation topography correction coefficient data set in this study is hosted at the National Tibetan Plateau/Third Pole Environment Data Center (10.11888/Atmos.tpdc.300784)^53^. There are three sets of files: (a) TCCSDR, (b) TCCDIF, and (c) TCCREF. The terrain correction coefficient data is stored in geotiff format with file names following the pattern “TCCSDR_lon_lat_LON_LAT_SZA_SAA_rr.tif”, “TCCDIF_lon_lat_LON_LAT_rr.tif”, and “TCCREF_lon_lat_LON_LAT_rr.tif”. Among them, lon, lat, LON, LAT, SZA, SAA and rr mean minimum longitude value of image range (°), minimum latitude value of image range (°), maximum longitude value of image range (°), maximum latitude value of image range (°), Solar Zenith Angle (°), Solar Azimuth Angle (°), and rr: spatial resolution, respectively (Table 3).

**Table 3:** Variable description in data naming.

Name	Description
TCCSDR	Terrain correction coefficient for solar direct radiation
TCCDIF	Terrain correction coefficient for diffused radiation
TCCREF	Terrain correction coefficient for adjacent surface reflected radiation
lon	Minimum longitude value of image range (°)
LON	Maximum longitude value of image range (°)
lat	Minimum latitude value of image range (°)
LAT	maximum latitude value of image range (°)
SZA	Solar Zenith Angle (°)
SAA	Solar Azimuth Angle (°)
rr	Spatial resolution

For example, a file named “TCCSDR_60_20_65_25_10_100_3 s.tif” represents the geotiff file that describes the TCCs data of solar direct radiation in the longitude range of 60–65°E and latitude range of 20–25°N when the SZA is 10° and the SAA is 100°. The TCCs can be calculated as ${\rm{TCCs}}=0.001\times {\rm{DN}}$, where DN is the digital quantized value. the real TCCs needs to be multiplied by the quantized value in the file by a coefficient of 0.001.

**Fig. 5:**
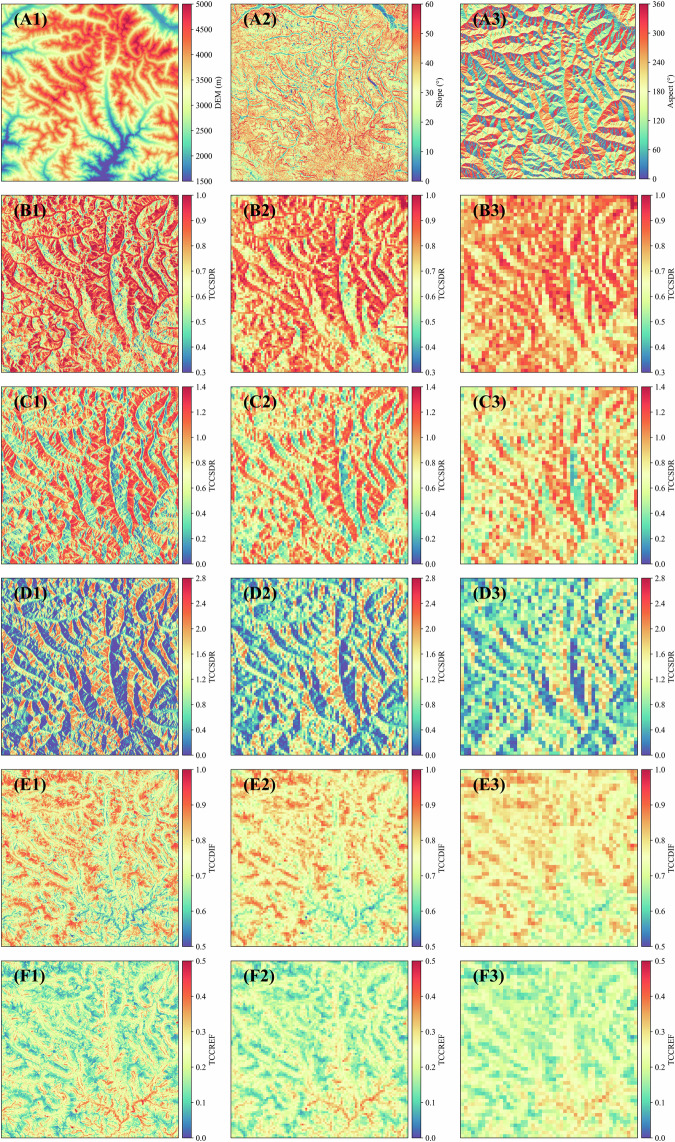
The spatial distribution maps of the TCCs and topographic features in the N28E096 region (consistent with region C in Fig. 1 and Table 1). The map is divided into several components: A1 represents the DEM, while A2 and A3 depict slope and aspect, respectively. B1-B3 show the TCCSDR spatial distribution when SZA is 20° and SAA is 90°, with spatial resolutions of 3s, 15 s and 30 s. C1- C3 and D1-D3 are consistent with the B1-B3, but the SZA are 45° and 70°, respectively; E1-E3 and F1-F3 depict the spatial distribution of TCCDIF and TCCREF, respectively.

And Fig. 5 shows the topography conditions and spatial distribution of TCCs in some areas used in the accuracy verification process of this article.

## Technical Validation

### Validate the terrain correction results of solar direct radiation using TCCSDR dataset

**Fig. 6:**
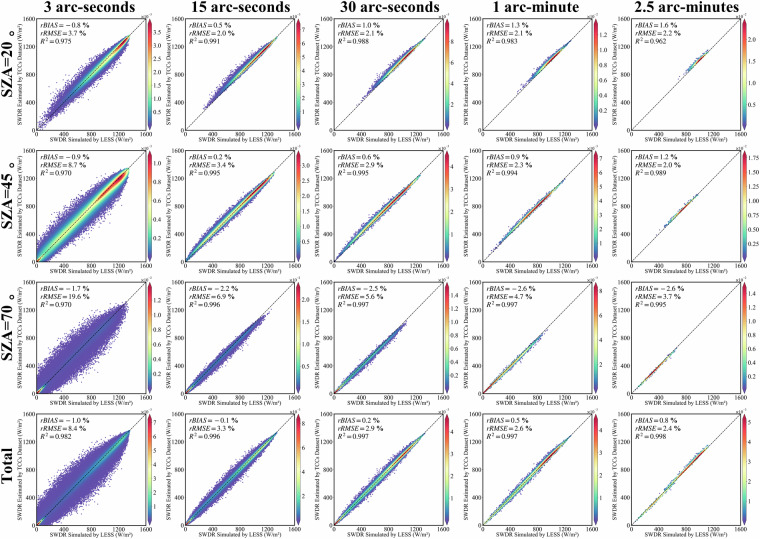
Validation of the terrain-corrected solar direct radiation by the TCCSDR in multiple spatial scales/SZAs based on LESS.

**Fig. 7:**
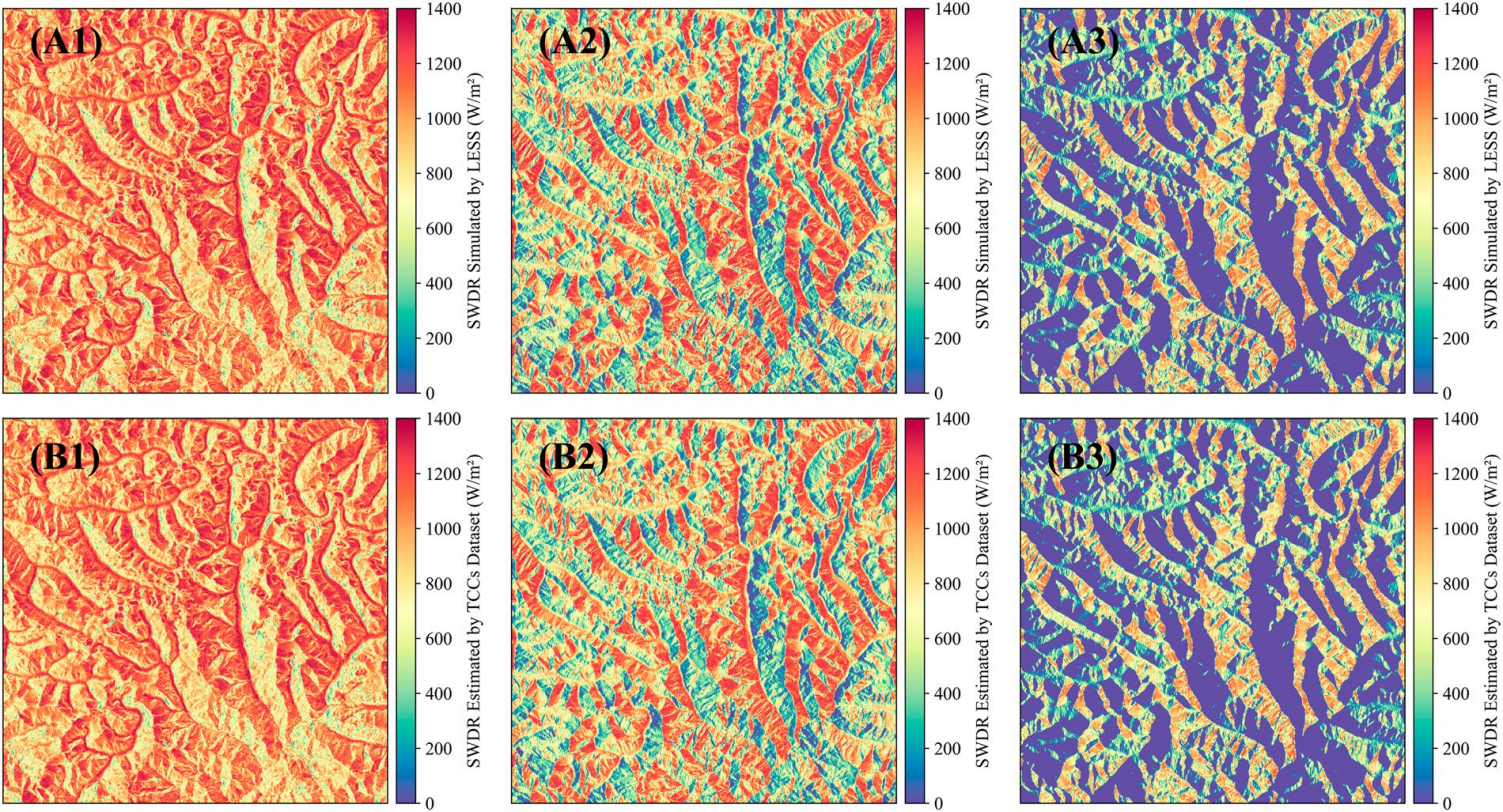
The spatial distribution of solar direct radiation received by the N28E096 region when the SAA is 90°, and the spatial resolution is 3 arc-seconds. The SZAs from left to right are 20°, 45° and 70° respectively. The first line is the result of LESS, and the second line is the result of TCCs.

To assess the correction results of TCCSDR for solar direct radiation, especially in applications related to vegetation biological processes and solar energy utilization, we conducted a validation by configuring LESS with a surface albedo of 0 as a control. The topographic correction results of TCCSDR under various spatial resolutions and SZAs are depicted in Fig. 6. The R^2^ values for all TCCSDR terrain correction outcomes exhibit a high level of agreement with the LESS simulation results, each exceeding 0.96. As the spatial resolution increases, the rRMSE consistently decreases. Notably, at 3 arc-seconds spatial resolution, the R^2^ is 0.982, with rRMSE at 8.4% and rbias at −1.0%. Figure 7 shows the SWDR results in mountainous areas simulated by LESS and corrected using TCCs at a spatial resolution of 3 arc-seconds. Meanwhile, at 15 arc-seconds spatial resolution, R^2^ stands at 0.996, with rRMSE at 3.3% and rbias at −0.1%. Further improvement is evident at 30 arc-seconds spatial resolution, where R^2^ reaches 0.997, with rRMSE at 2.9% and rbias at 0.2%. At 1 arc-minute spatial resolution, the R^2^ is 0.997, with rRMSE at 2.6% and rbias at 0.5%. Lastly, at 2.5 arc-minutes spatial resolution, R^2^ registers at 0.998, accompanied by rRMSE at 2.4% and rbias at 0.8%. Consequently, TCCSDR at varying spatial resolutions demonstrates a reliably high level of accuracy, effectively catering to the diverse data requirements for terrain correction of direct solar radiation.

### Validate the terrain correction results of diffuse radiation using TCCDIF dataset

**Fig. 8:**
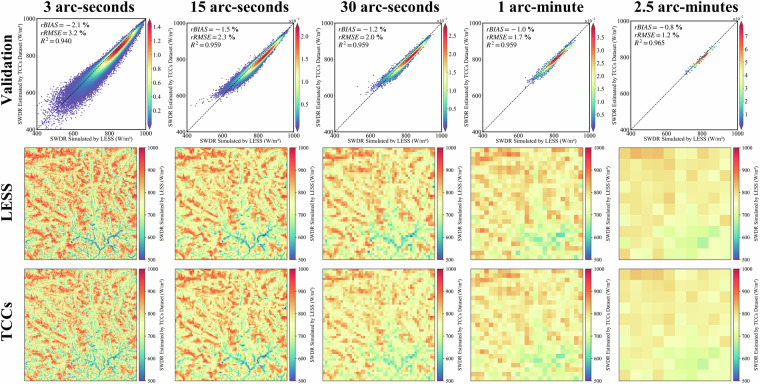
The first row is validation of the terrain-corrected diffuse radiation by the TCCDIF in multiple spatial scales based on LESS; The second row is the spatial distribution of SWDR received by the N28E096 region simulated by LESS, with a spatial resolution of 3 arc-seconds. The third row is the result of TCCDIF.

Similar to the validation for solar direct radiation, we evaluated the topographic correction results of TCCDIF for diffuse radiation by setting the albedo to 0. TCCDIF effectively performs topographic correction for diffuse radiation across five spatial resolutions, with accuracy consistently improving as the spatial resolution coarsens (Fig. 8). At a 3 arc-second spatial resolution, the results yield an R^2^ of 0.940, a rRMSE of 3.2%, and a rbias of −2.1%. Figure 8 also shows the SWDR results in mountainous areas simulated by LESS and corrected using TCCs at a spatial resolution of 3 arc-seconds. Progressing to a 15 arc-second spatial resolution, the R^2^ increases to 0.959, with rRMSE at 2.3% and rbias at −1.15%. At a 30 arc-second spatial resolution, the R^2^ reaches 0.959, accompanied by rRMSE at 2.0% and rbias at −1.2%. For a 1 arc-minute spatial resolution, the R^2^ stands at 0.959, with rRMSE at 1.7% and rbias at −1.0%. Lastly, at a 2.5 arc-minutes spatial resolution, the R^2^ reaches 0.965, with rRMSE at 1.2% and rbias at −0.8%. Consequently, TCCDIF efficiently converts diffuse radiation assumed on a flat surface into the actual results received on the mountainous surface, demonstrating effective terrain correction accuracy.

### Validate the terrain correction results of SWDR under clear sky conditions using TCCs dataset

The SWDR received by the surface under clear sky conditions primarily comprises solar direct radiation and reflected radiation from adjacent terrain. Consequently, in this segment, we set the surface albedo in the LESS to 0.2 (representing grass) and 0.8 (representing snow). Notably, as sky diffuse radiation contributes insignificantly to the radiation received by the surface under clear sky conditions, we set the sky diffuse radiation to 0.

At a spatial resolution of 3 arc-seconds, for low surface albedo (grass), the rbias is 3.4%, the rRMSE is 20.9%, and the R^2^ is 0.883. For high surface albedo (snow), the rbias is 3.9%, the rRMSE is 23.0%, and the R^2^ is 0845. Coarsening the spatial resolution leads to a slight increase in rbias, a marginal decrease in rRMSE, and an increase in R^2^. When the spatial resolution is further coarsened to 2.5 arc-minutes, the terrain correction results for SWDR using TCCs show an rbias of 5.1%, a RMSE of 7.8%, and a R^2^ of 0.989 for low surface albedo representing grass. For high surface albedo representing snow, the rbias is 4.9%, the rRMSE is 8.3%, and the R^2^ is 0.979.

**Fig. 9:**
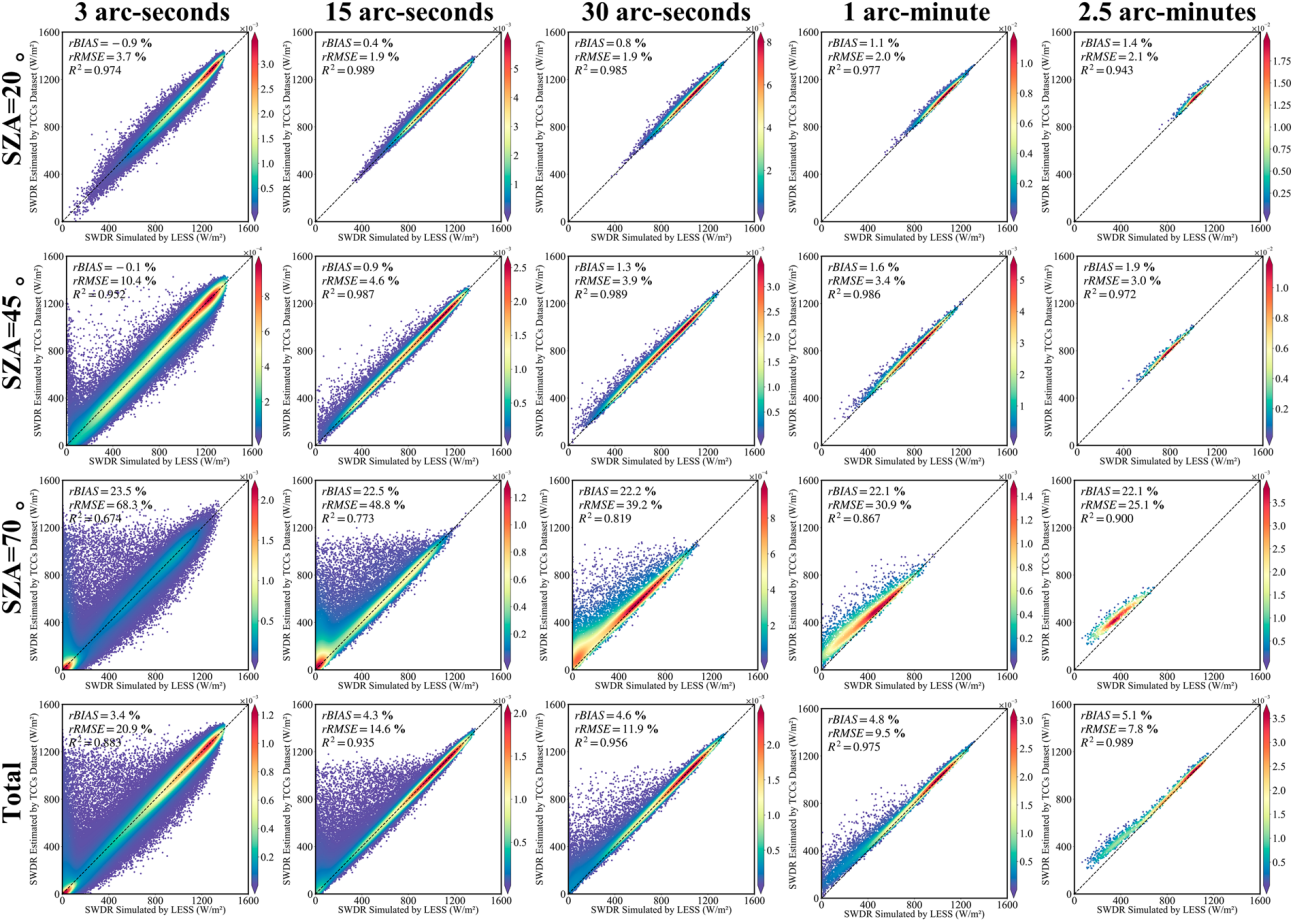
Validation of the terrain-corrected solar direct radiation by the TCCSDR in multiple spatial scales/SZAs based on LESS, in the grass scenario.

**Fig. 10:**
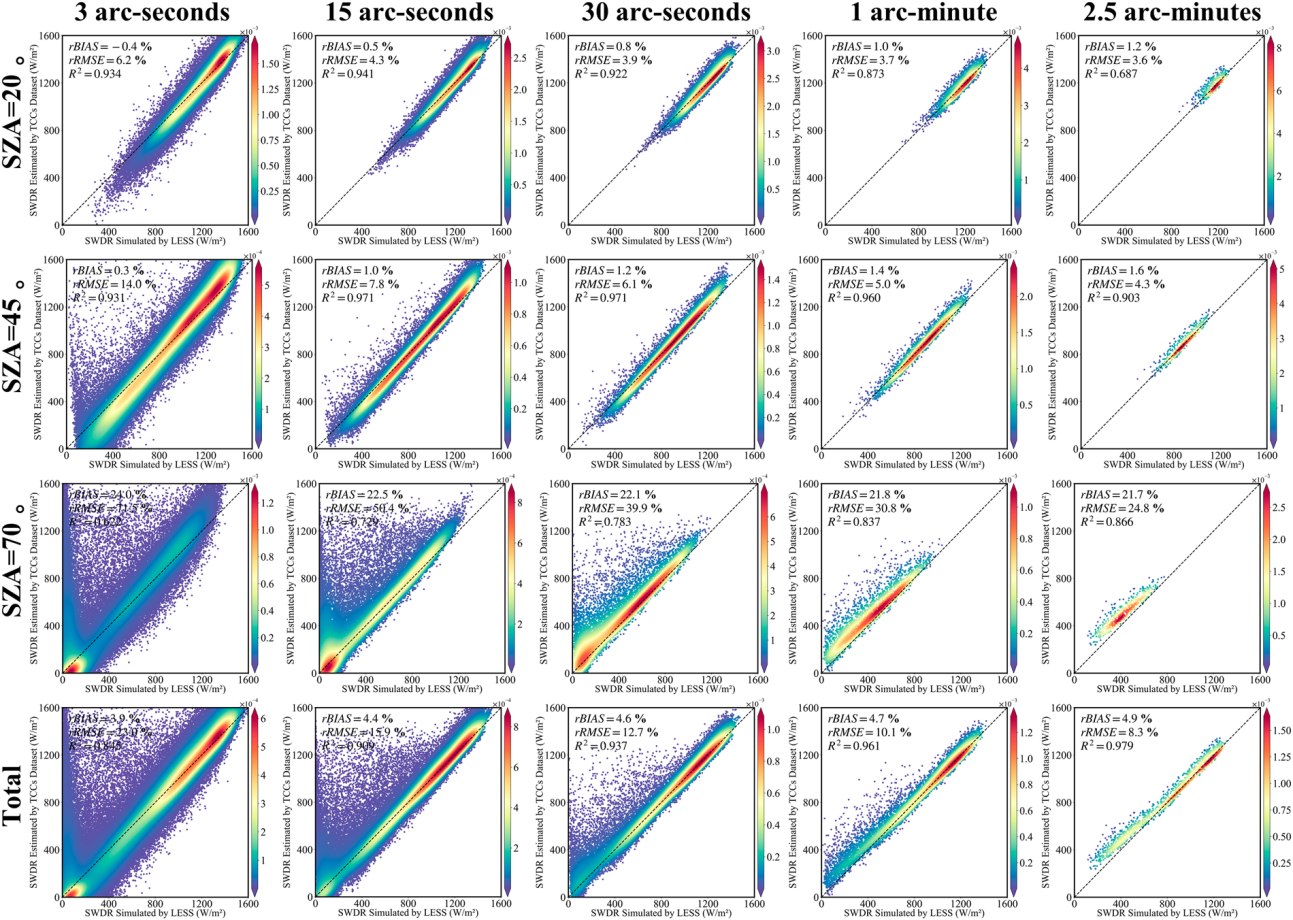
Validation of the terrain-corrected solar direct radiation by the TCCSDR in multiple spatial scales/SZAs based on LESS, in the snow scenario.

The outcomes of the topographic correction for SWDR using TCCs under different SZAs reveal consistent validation results for SZAs of 20° and 45°, both demonstrating excellent accuracy. However, the accuracy diminishes as the SZA increases to 70°. When the spatial resolution is 2.5 minutes in the grass scenario, with a SZA of 20°, the rbias is 1.4%, the rRMSE is 2.1%, and the R² is 0.943 (Fig. 9). For an SZA of 45°, the rbias is 1.9%, the rRMSE is 3.0%, and the R² is 0.972. When the SZA is 70°, the rbias increases to 22.1%, the rRMSE to 25.1%, and the R² decreases to 0.9. In the snow scenario, at the same spatial resolution of 2.5 minutes, when the SZA is 20°, the rbias is 1.2%, the rRMSE is 3.6%, and the R² is 0.687. For an SZA of 45 degrees, the rbias is 1.6%, the rRMSE is 4.3%, and the R² is 0.903. At an SZA of 70 degrees, the rbias is 21.7%, the rRMSE is 24.8%, and the R² is 0.866 (Fig. 10).

In comparison with the results of solar direct radiation, the accuracy of SWDR terrain correction results under clear sky conditions has experienced a decline. This decline in accuracy is attributed to the characterization method of reflected radiation used in this article is still controversial. Chu *et al*.^54^ argue that, at the regional average scale, the complementary value of the sky view factor effectively depicts terrain reflection and aligns with the ray tracing model. Conversely, Shi *et al*.^27^ point out that this method introduces additional errors and fails to provide a precise estimate of the contribution of reflections from adjacent terrain. In spite of the ongoing debate, the method used in this study for terrain correction of terrain reflection is retained due to its simplicity and parametric usability, especially when compared to other methods for calculating adjacent terrain reflection. Users can decide whether to use this method to calculate SWDR terrain reflection according to their own needs.

### Validate the terrain correction results of SWDR under cloudy sky condition using TCCs dataset

In contrast to the clear sky condition, under cloudy conditions, solar direct radiation struggles to reach the surface directly due to cloud cover under cloudy-sky conditions. The SWDR received by the surface mainly comprises diffuse radiation from the sky and reflected radiation from the adjacent surface. Therefore, when simulating SWDR received by the surface in cloudy skies using LESS, the solar direct radiation is set to 0. The SWDR received by the surface under surface reflectivity representing grass and snow is then simulated. The results of terrain correction using TCCs under cloudy conditions demonstrate consistent performance for both grass and snow.

**Fig. 11:**
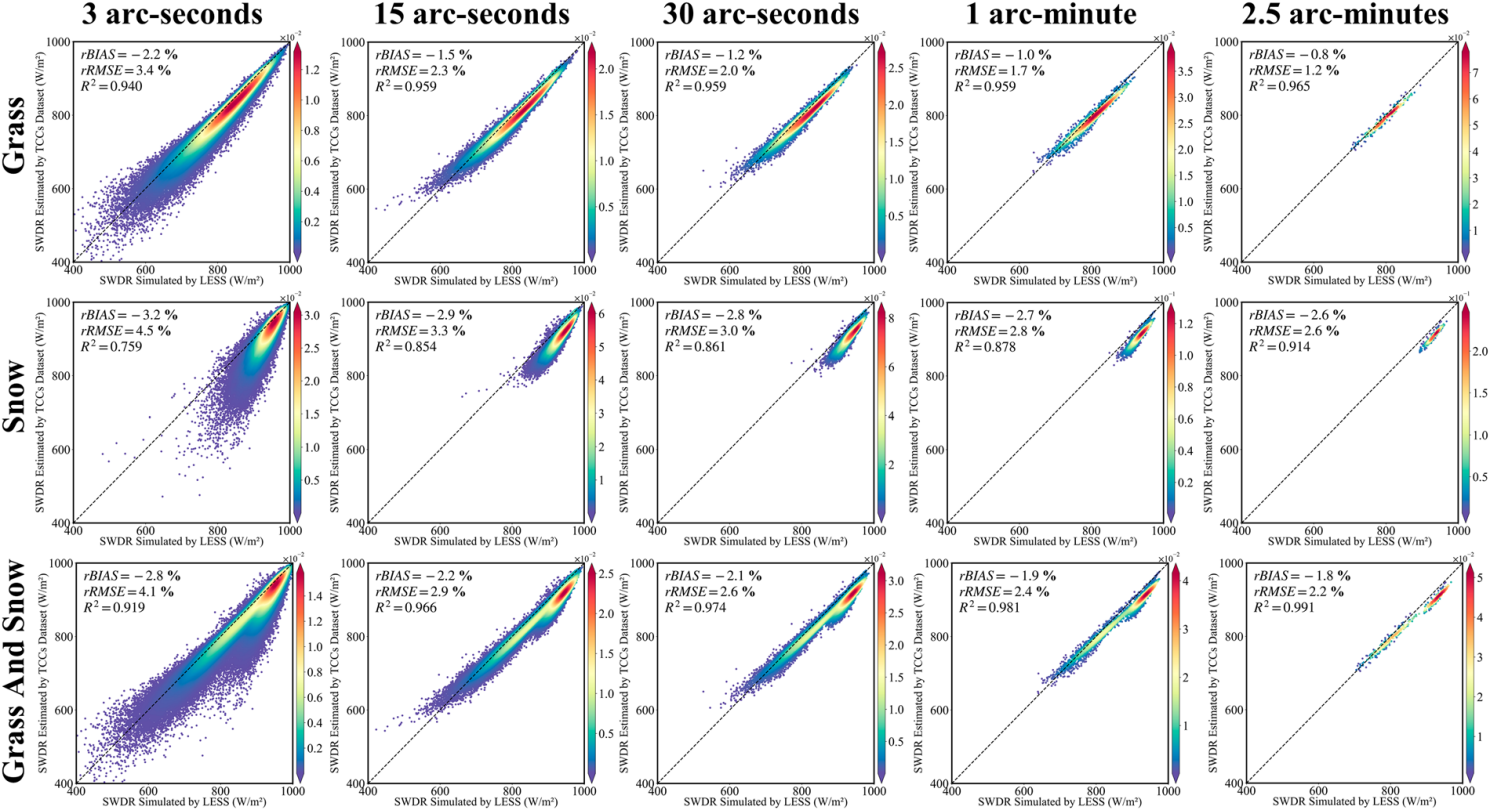
Validation of the terrain-corrected SWDR by the TCCs in multiple spatial scales based on LESS, under cloudy sky condition.

At varying spatial resolutions, the performance metrics show the effectiveness of TCCs in terrain correction (Fig. 11). In particular, at a spatial resolution of 3 arc-seconds, the rbias is −2.8%, rRMSE is 4.1%, and R^2^ is 0.919. As the spatial resolution increases to 15 arc-seconds, the rbias improves to −2.2%, rRMSE decreases to 2.9%, and R^2^ stands at 0.966. Further improvement is observed at 30 arc-seconds spatial resolution, with an rbias of −2.1%, rRMSE of 2.6%, and an impressive R^2^ of 0.974. Similarly, at 1 arc-minute spatial resolution, the rbias is −1.9%, rRMSE is 2.4%, and R^2^ is 0.981. Finally, at 2.5 arc-minutes spatial resolution, the rbias is −1.8%, rRMSE is 2.2%, and R^2^ reaches 0.991.

It is worth noting that, under cloud-sky conditions, the influence of SWDR in mountainous regions manifests in three distinct parts. Firstly, the presence of mountain occlusion, epitomized by the ${V}_{d}$, impedes the reception of diffuse radiation within the hemispheric space by the mountainous terrain. Consequently, notwithstanding a diffuse radiation of 1000 W/m^2^ from the sky, regions other than open expanses such as mountain peaks and plains receive scattered radiation lower than 1000 W/m^2^. Secondly, the rugged and intricate nature of mountainous terrain contributes to the phenomenon. Although the total radiation flux received by a pixel remains constant, the actual surface area of mountainous terrain exceeds that of flat surfaces, thereby further weaken the unit area density of radiant flux (W/m^2^). This effect is encapsulated by the area ratio factor (*t*) in the Eq. 13. Thirdly, mountainous surfaces receive reflected radiation from adjacent surfaces, augmenting the SWDR received. In synthesis, both the first and second parts attenuate the SWDR received by mountainous surfaces. While the third part increases the SWDR received, it is constricted by the preceding two factors. Hence, despite the robust reflection flux on snow-cover surface observed, the radiation flux density received on the surface of mountainous regions remains inferior to that on flat terrain.

It is essential to note that our validation focused on the terrain correction effect of TCCs under ideal clear and cloudy conditions. Real atmospheric conditions often fall somewhere between these two extremes. Although we haven’t explicitly verified the accuracy of terrain correction for SWDR composed of direct and diffuse radiation in varying proportions, it can be inferred that its accuracy is a proportional weighting of the clear sky and cloudy sky condition accuracy results discussed in this article.

As indicated by the validation results outlined above, the correction outcomes achieved with our TCCs dataset exhibit a remarkable alignment with the results obtained through ray tracing-based three-dimensional radiation transfer simulations. This implies that our product can significantly assist users in efficiently and accurately implementing SWDR terrain correction in the Pan-Third Pole region. However, it’s important to note that in our current study, SZA and SAA are examined at discrete intervals of 5°. In real applications, these angles are continuous rather than discrete. Therefore, when utilizing our product, users still need to interpolate the TCCs values based on the actual SZA and SAA (e.g., the nearest neighbour method). While this interpolation process may introduce some error into the topographic correction of instantaneous SWDR, upscaling the instantaneous results to a daily scale can effectively mitigate its impact. In our forthcoming research endeavours, we are committed to refining the algorithm and developing data products with smaller SZA and SAA intervals.

## Data Availability

All the codes used in this study to construct the dataset were written in the Interactive Data Language (IDL) and will be openly available at https://github.com/YuyangXian/TCC.git.
